# Use of multi-trait and random regression models to identify genetic variation in tolerance to porcine reproductive and respiratory syndrome virus

**DOI:** 10.1186/s12711-017-0312-7

**Published:** 2017-04-19

**Authors:** Graham Lough, Hamed Rashidi, Ilias Kyriazakis, Jack C. M. Dekkers, Andrew Hess, Melanie Hess, Nader Deeb, Antti Kause, Joan K. Lunney, Raymond R. R. Rowland, Han A. Mulder, Andrea Doeschl-Wilson

**Affiliations:** 1 The Roslin Institute & R(D)SVS, University of Edinburgh, Edinburgh, Midlothian, UK; 2 Animal Breeding and Genomics Centre, Wageningen University and Research, PO Box 338, 6700 AH Wageningen, The Netherlands; 3 School of Agriculture Food and Rural Development, Newcastle University, Newcastle upon Tyne, NE1 7RU UK; 4 Department of Animal Science, Iowa State University, Ames, IA 50011 USA; 5 Genus plc, 100 Bluegrass Commons Blvd. Suite 2200, Hendersonville, TN 37075 USA; 6 Biometrical Genetics, Natural Resources Institute Finland, 00790 Jokioinen, Finland; 7 Animal Parasitic Diseases Laboratory, USDA, Beltsville, MD 20705 USA; 8 College of Veterinary Medicine, Kansas State University, Manhattan, KS 66506 USA

## Abstract

**Background:**

A host can adopt two response strategies to infection: resistance (reduce pathogen load) and tolerance (minimize impact of infection on performance). Both strategies may be under genetic control and could thus be targeted for genetic improvement. Although there is evidence that supports a genetic basis for resistance to porcine reproductive and respiratory syndrome (PRRS), it is not known whether pigs also differ genetically in tolerance. We determined to what extent pigs that have been shown to vary genetically in resistance to PRRS also exhibit genetic variation in tolerance. Multi-trait linear mixed models and random regression sire models were fitted to PRRS Host Genetics Consortium data from 1320 weaned pigs (offspring of 54 sires) that were experimentally infected with a virulent strain of PRRS virus to obtain genetic parameter estimates for resistance and tolerance. Resistance was defined as the inverse of within-host viral load (VL) from 0 to 21 (VL_21_) or 0 to 42 (VL_42_) days post-infection and tolerance as the slope of the reaction-norm of average daily gain (ADG_21_, ADG_42_) on VL_21_ or VL_42_.

**Results:**

Multi-trait analysis of ADG associated with either low or high VL was not indicative of genetic variation in tolerance. Similarly, random regression models for ADG_21_ and ADG_42_ with a tolerance slope fitted for each sire did not result in a better fit to the data than a model without genetic variation in tolerance. However, the distribution of data around average VL suggested possible confounding between level and slope estimates of the regression lines. Augmenting the data with simulated growth rates of non-infected half-sibs (ADG_0_) helped resolve this statistical confounding and indicated that genetic variation in tolerance to PRRS may exist if genetic correlations between ADG_0_ and ADG_21_ or ADG_42_ are low to moderate.

**Conclusions:**

Evidence for genetic variation in tolerance of pigs to PRRS was weak when based on data from infected piglets only. However, simulations indicated that genetic variance in tolerance may exist and could be detected if comparable data on uninfected relatives were available. In conclusion, of the two defense strategies, genetics of tolerance is more difficult to elucidate than genetics of resistance.

**Electronic supplementary material:**

The online version of this article (doi:10.1186/s12711-017-0312-7) contains supplementary material, which is available to authorized users.

## Background

Infectious challenges in domestic livestock do not only raise health and welfare concerns, but also have detrimental effects on livestock production. The impact of infections on an animal’s productive performance is controlled by two alternative (albeit not mutually exclusive) host traits that may be amenable to genetic improvement: resistance and tolerance. Resistance is defined as the ability of a host to prevent pathogen entry or inhibit replication of the pathogen, whereas tolerance refers to the ability of a host to limit the impact of infection on health or performance without interfering with the pathogen life-cycle per se [1]. Thus, animals with greater resistance are expected to harbor fewer pathogens that can lead to loss in performance. In contrast, animals with greater tolerance may harbor a high within-host pathogen load but are able to prevent or repair the damage of infection on health and performance [2, 3]. To date, most efforts to control infectious disease have targeted primarily improvement of host resistance. More recently, the focus has expanded towards boosting host tolerance as an alternative means to counteract the detrimental impact of infection on health and performance [4, 5]. However, little is known about the extent to which tolerance is genetically controlled and thus suitable for genetic improvement.

Porcine reproductive and respiratory syndrome (PRRS) is an endemic virus, which causes one of the most devastating swine diseases worldwide. PRRS causes considerable reduction in the growth rate of piglets, with estimates ranging from 10 to 20%, depending on pig breed and virus strain [6], and results in production losses amounting to an annual cost of $493.57 million to the U.S. swine industry alone [7]. Since vaccination has been largely unsuccessful [8], genetic solutions to PRRS have gained increased attention [9–11]. Recent large-scale PRRSV challenge studies carried out by the PRRS Host Genetics Consortium (PHGC) have demonstrated considerable genetic variation in resistance of pigs to PRRSV (virus) infection, as well as in weight gain of infected piglets [10, 12, 13]. Furthermore, genetic correlations between resistance and weight gain were shown to be positive and strong (ranging from 0.57 to 0.75 for two different PRRSV strains) [10], indicating that selection for improved resistance is expected to simultaneously improve growth under infection and vice versa. However, it is not currently known whether pigs also differ genetically in their tolerance to PRRSV infection, or whether pigs with greater genetic resistance to PRRSV are also genetically more tolerant to PRRS.

**Fig. 1:**
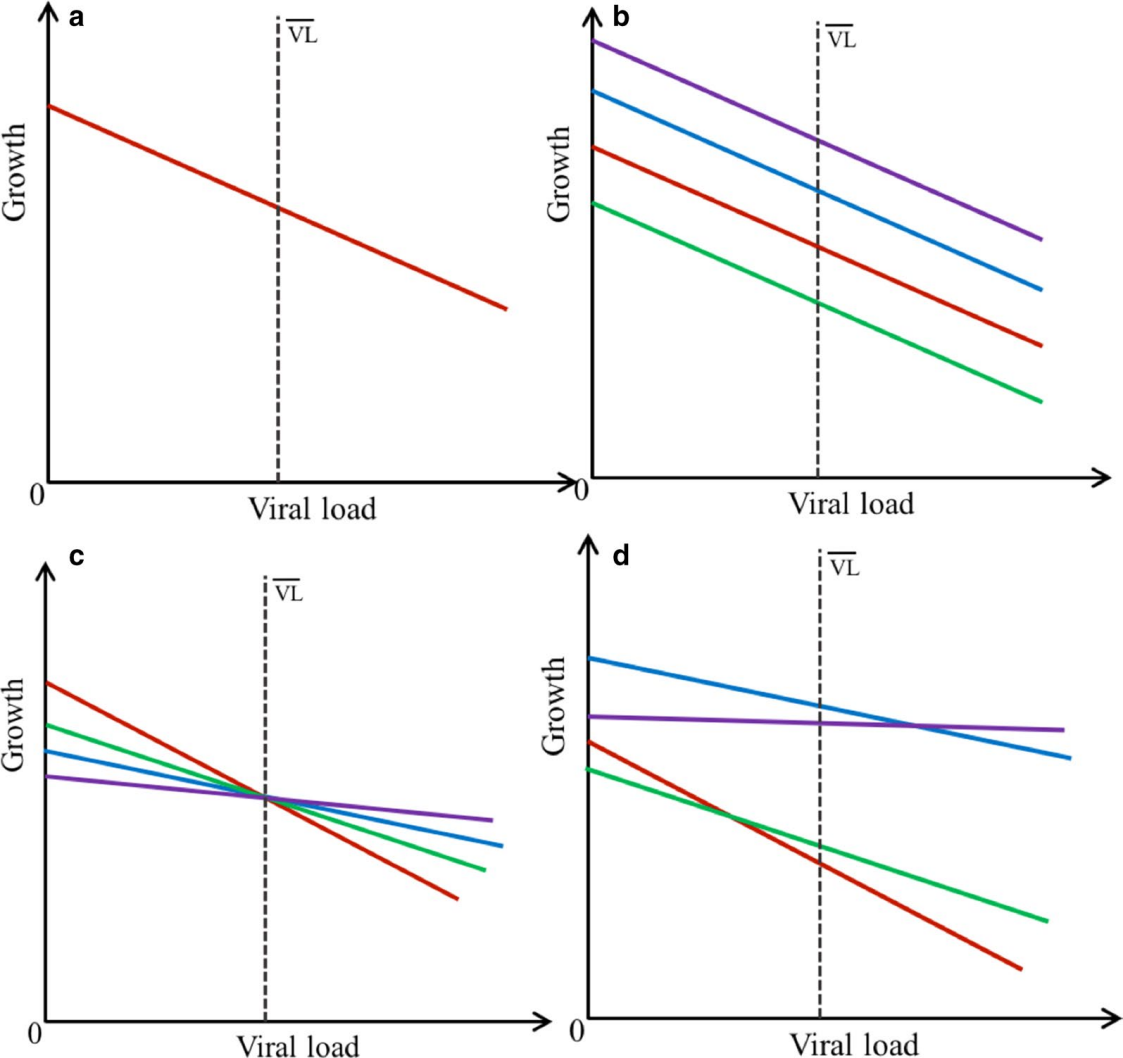
Graphical illustration of reaction norms for analysis of tolerance. Mean VL is indicated by the *stippled line* in each graph. Each line corresponds to one of four hypothetical sires. **a** Null model, where all sires have equal tolerance and equal overall growth level. As such, there is only one (average) tolerance slope. **b** Reaction-norms of sires with equal tolerance. Sires differ in intercept (growth where VL = 0) and level (growth at mean VL), but have equal tolerance slopes. No re-ranking of sires occurs between growth associated with low and high VL, and genetic correlation between intercept and level is 1.00. **c** Reaction norms of sires with variation in intercept and tolerance slopes, but no variation in level. Re-ranking of sires occurs depending on whether offspring harbor low or high VL, respectively, as indicated by crossing over of lines before and after mean VL. **d** Reaction norms of sires where variation occurs at intercept, level and tolerance slope. Sire re-ranking occurs between low and high VL, and genetic correlation between intercept and level is below one

Resistance can be measured as the inverse of within-host pathogen load, whereas tolerance is related to the degree to which performance is reduced by infectious pathogens. Tolerance is mathematically defined as a reaction-norm of performance with respect to pathogen load [2, 14]. Assuming a linear relationship, reaction-norms can be modelled by a linear regression of performance against pathogen load, where the regression slope provides a measure of tolerance (Fig. 1). Thus, a slope of 0 indicates complete tolerance, while a more negative slope indicates lower tolerance. Statistically significant differences in reaction-norm slopes associated with, e.g., different breeds or families are indicative of genetic variation in tolerance. For outbred populations, tolerance slopes for groups of related individuals can be estimated by random regression models, which provide estimates for genetic variance of tolerance and for genetic variance in host performance as a function of pathogen load when combined with pedigree or genomic information [15]. However, due to the large amount of data required to obtain unbiased variance estimates for reaction norm slopes [15–17], very few studies have applied this methodology to gain insight into the genetic basis of tolerance in outbred populations [18]. The PRRS Host Genetics Consortium (PHGC) data, which provide simultaneous measures of growth and viral load for over 1500 pedigreed pigs infected with the same PRRS virus load offer a unique opportunity to estimate genetic parameters for tolerance.

The main aim of this study was to determine whether pigs that were previously found to differ genetically in resistance to a virulent strain of PRRSV also differ genetically in tolerance. Furthermore, by augmenting the data with simulated data, novel insights into data requirements to accurately estimate genetic variance in tolerance using random regression models were obtained.

## Methods

### Infection experiment and data

Data were provided by the PRRS Host Genetics Consortium (PHGC) from nine different PRRSV challenge trials with an identical infection protocol [9, 10], which included 1569 pigs supplied by various commercial breeding companies, as outlined in Table 1.

**Table 1:** Animal, pedigree and breed composition of the PHGC trials

Trial	Number of animals	Number of sires	Number of dams	Breed cross
1	174	6	70	LW × LR
2	164	10	72	LW × LR
3	115	7	47	LW × LR
4	191	6	33	Duroc × LW/LR
5	182	10	38	Duroc × LR/LW
6	109	26	53	LR × LR
7	186	6	27	Pietrain × LW/LR
8	158	15	43	Duroc × LW/LR
15	166	11	49	Pietrain × LW

*LW* large white breed, *LR* landrace breed

All experimental protocols for these trials were approved by the Kansas State University Institutional Animal Care and Use Committee. In each trial, approximately 200 commercial crossbred piglets were transferred from high health farms at weaning age (mean age = 26 days, range = 17 to 32 days) to a research facility at Kansas State University. The source farms were controlled and found to be free of PRRSV, *Mycoplasma hyopneumoniae*, and swine influenza virus. Pigs were randomly placed in pens of 10 to 15 individuals. Following a 7-day acclimation period, pigs between 17 and 32 days of age were experimentally infected both intramuscularly and intranasally with 10^5^ (TCID50) of NVSL-97-7985, a highly virulent PRRSV isolate [19]. Body weight (BW) and blood samples were collected at 0, 7, 14, 21, 28, 35 and 42 days-post-infection (dpi). Pigs were then euthanized at 42 dpi and ear notches were collected for genotyping. Trials 7 and 8 were terminated at 35 dpi because of facility availability. Estimates for average daily gain (ADG) from 0 dpi until day of measurement were obtained by dividing the difference in body weight between the day of observation and 0 dpi by the corresponding time period. Note that neither measurements of ADG for these pigs prior to infection, nor ADG measurements for non-infected relatives were available.

Serum viremia, which was measured by using a semi-quantitative TaqMan PCR assay for PRRSV RNA, provided repeated (bi-weekly up to 14 dpi, then weekly) measures for log_10_-transformed qPCR viremia, as described in Boddicker et al. [12, 13, 20]. Mathematical functions were previously fitted to these log_10_-transformed viremia measures to smooth the data and to obtain continuous viremia estimates over the 42-day observation period [21]. As outlined in Islam et al. [21], the uni-modal Woods function and the extended bi-modal Woods function provided a good fit to the individual’s data with either uni-modal ($y\left( t \right) = a_{1} t^{{b_{1} }} e^{{ - c_{1} t}}$) (~67%) or bi-modal $\left(y\left( t \right) = a_{1} t^{{b_{1} }} e^{{ - c_{1} t}} + { \hbox{max} }\left( {0,a_{2} \left( {t - t_{0} } \right)^{{b_{2} }} e^{{ - c_{2} }} \left( {t - t_{0} } \right)} \right)\right)$ (~33%) viremia profiles, respectively, with strong correlations between model predictions of VL and actual viremia measures (genetic and phenotypic correlation estimates were 0.98 ± 0.03 and 0.90 ± 0.01, respectively) [10].

Across all trials, 198 pigs died before 42 dpi. PRRS was identified as the primary cause of mortality, except for trial 6, for which mortality was higher (46% by 42 dpi) and was potentially caused by secondary bacterial infections [13]. These pigs were included in the analyses until their time of death.

Only offspring from sires with more than 10 progeny with phenotypes were considered in this study to reduce the risk of bias in tolerance estimates [15]. As such, the number of animals included was 1320 from 0 to 21 dpi and 1001 from 0 to 42 dpi, all originating from 54 sires.

Pedigree information and genomic information using genotypes from Illumina’s Porcine SNP60 Beadchip v.1 [22], was available for all pigs. The pedigree-based numerator relationship matrix (${\mathbf{A}}$) and genomic relationship ${\mathbf{G}}$-matrix (${\mathbf{G}}_{\text{m}}$), were constructed in ASReml 3.0 [23] using the VanRaden method for all animals used in the analysis. For the ${\mathbf{G}}$-matrix, single nucleotide polymorphisms (SNPs) that were fixed in a trial were removed. Trials 1, 2, and 3 had the most extensive pedigree information, with pedigree data up to two generations back, while the rest of the trials only had sire and dam recorded. As such, there were no relationships between animals in different trials, except for trials 1, 2, and 3, which consisted of animals from consecutive parities of the same breeding company (Table 1). Pedigree was corrected using parental genotypes for all trials, as described by Boddicker et al. [13] and Hess et al. [10]. The ${\mathbf{G}}$-matrix was constructed using the VanRaden method [24], and included relationships between animals across trials regardless of breed, as outlined by Hess et al. [10]. The ${\mathbf{A}}$-matrix was used for all the following statistical models, unless otherwise noted.

### Resistance, tolerance, and performance without infection

Resistance is often quantified by a measure of within-host pathogen load, whereby lower pathogen load reflects higher host resistance [2, 5, 16]. In this study, resistance to PRRS was defined as the inverse of serum viral load, whereby VL_42_ represents the cumulative log-transformed viral load from 0 to 42 dpi from the Wood’s curve. Since viremia had decreased to undetectable levels within 21 to 28 dpi for a large proportion of pigs, cumulative viral load (and thus resistance) was not only calculated for the entire observation period from 0 to 42 dpi, but also for the period from 0 to 21 dpi. This represents the acute phase of infection and yields two indicator traits for resistance (VL_21_ and VL_42_).

In this study, tolerance was assessed by regressing performance measures (i.e. ADG_21_ or ADG_42_ on the y-axis) on pathogen load (i.e. VL_21_ or VL_42_, respectively on the x-axis). The regression of ADG_42_ on VL_21_ was also evaluated to account for the possibility of a time-lag in growth response with respect to changes in pathogen load.

Growth performance of an infected individual is likely to depend on both their response to infection and performance in the absence of infection. Performance in absence of infection (i.e. when pathogen load is equal to zero), commonly denoted in the tolerance literature as vigor [25], constitutes the intercept of the linear reaction-norms (Fig. 1). Previous simulation studies indicated that performance measures in the absence of infection are important to obtain unbiased tolerance slope estimates [15]. However, information on performance of the PRRSV challenged pigs in absence of infection was not available in this study.

Two approaches were adopted to overcome this lack of performance measures without infection. (1) In line with the standard approach of quantitative genetic studies of reaction-norms, the origin of the explanatory variable VL was shifted to the mean VL; this ‘shifted intercept’ for ADG is referred to as the ‘level’, in contrast to vigor [17, 26, 27] (Fig. 1); note that this approach does not provide accurate information about the genetic relationship between tolerance and vigor, as the genetic correlation between level and slope is not equal to the genetic correlation between performance at VL = 0 and slope [28, 29]. Furthermore, individual body weight at the start of the infection (BW_0_) was included as a fixed covariate in the corresponding statistical models to partially account for differences in vigor. (2) To gain better insight into data requirements for accurately estimating genetic parameters for tolerance, and about how these estimates depend on the genetic relationship between growth in absence or presence of infection, growth records of infected pigs were augmented with simulated growth records of non-infected half-siblings, as outlined in step 4 below.

### Statistical analyses

All statistical analyses were carried out using ASReml 3.0 [23]. Random regression reaction-norm models have been found to provide biased estimates if data requirements to disentangle intercept from slope are not met [15, 17, 30], thus a stepwise approach was adopted: (Step 1) multi-trait animal models were used to estimate the genetic relationship between resistance and growth under infection; (Step 2) multi-trait models were used to provide evidence for genetic variation in tolerance of pigs to PRRS based on the genetic correlation between growth associated with low and high VL, respectively; (Step 3) a univariate random regression model was applied to obtain estimates for genetic variance in tolerance; and (Step 4) data were augmented using simulated performance in the absence of infection (ADG_21_^0^ or ADG_42_^0^), with increasing simulated genetic correlation from weak to strong between ADG_21_^0^ and ADG_21_ or ADG_42_^0^ and ADG_42_, respectively. The random regression models from Step 3 were adapted to include variation in ADG_21_^0^ or ADG_42_^0^.

#### Step 1: multi-trait models to estimate the genetic relationship between resistance and performance prior to and post infection

Our first step in analyzing variation in growth under infection was to estimate heritabilities and correlations between VL and growth in absence of and post-infection with PRRSV using the following trivariate animal model:

1 $$\left[ {\begin{array}{*{20}c} {{\mathbf{y}}_{1} } \\ {\begin{array}{*{20}c} {{\mathbf{y}}_{2} } \\ {{\mathbf{y}}_{3} } \\ \end{array} } \\ \end{array} } \right] = \left[ {\begin{array}{*{20}c} {{\mathbf{X}}_{1} } & 0 & 0 \\ 0 & {{\mathbf{X}}_{2} } & 0 \\ 0 & 0 & {{\mathbf{X}}_{3} } \\ \end{array} } \right]\left[ {\begin{array}{*{20}c} {{\mathbf{b}}_{1} } \\ {\begin{array}{*{20}c} {{\mathbf{b}}_{2} } \\ {{\mathbf{b}}_{3} } \\ \end{array} } \\ \end{array} } \right] + \left[ {\begin{array}{*{20}c} {{\mathbf{Z}}_{1} } & 0 & 0 \\ 0 & {{\mathbf{Z}}_{2} } & 0 \\ 0 & 0 & {{\mathbf{Z}}_{3} } \\ \end{array} } \right]\left[ {\begin{array}{*{20}c} {{\mathbf{a}}_{1} } \\ {\begin{array}{*{20}c} {{\mathbf{a}}_{2} } \\ {{\mathbf{a}}_{3} } \\ \end{array} } \\ \end{array} } \right] + \left[ {\begin{array}{*{20}c} {{\mathbf{U}}_{1} } & 0 & 0 \\ 0 & {{\mathbf{U}}_{2} } & 0 \\ 0 & 0 & {{\mathbf{U}}_{3} } \\ \end{array} } \right]\left[ {\begin{array}{*{20}c} {{\mathbf{p}}_{1} } \\ {\begin{array}{*{20}c} {{\mathbf{p}}_{2} } \\ {{\mathbf{p}}_{3} } \\ \end{array} } \\ \end{array} } \right] + \left[ {\begin{array}{*{20}c} {{\mathbf{M}}_{1} } & 0 & 0 \\ 0 & {{\mathbf{M}}_{2} } & 0 \\ 0 & 0 & {{\mathbf{M}}_{3} } \\ \end{array} } \right]\left[ {\begin{array}{*{20}c} {{\mathbf{l}}_{1} } \\ {\begin{array}{*{20}c} {{\mathbf{l}}_{2} } \\ {{\mathbf{l}}_{3} } \\ \end{array} } \\ \end{array} } \right] + \left[ {\begin{array}{*{20}c} {{\mathbf{e}}_{1} } \\ {\begin{array}{*{20}c} {{\mathbf{e}}_{2} } \\ {{\mathbf{e}}_{3} } \\ \end{array} } \\ \end{array} } \right],$$

where ${\mathbf{y}}_{1}$, ${\mathbf{y}}_{2}$ and ${\mathbf{y}}_{3}$ are vectors of phenotypes for body weight at the start of infection (BW_0_) (${\mathbf{y}}_{1}$), ADG_21_ or ADG_42_ (${\mathbf{y}}_{2}$), and VL_21_ or VL_42_ (${\mathbf{y}}_{3}$), respectively; ${\mathbf{b}}_{1} ,$ ${\mathbf{b}}_{2}$ and ${\mathbf{b}}_{3}$ are the vectors of the fixed effects for the interaction of experimental trial and parity of the dam when offspring were born (trial-by-parity), sex of the offspring, and age at start of experimental infection, which was fitted as a fixed covariate. Note that no breed effect was included in the model since trial and breed effects were fully confounded in this experiment. To account for differences between viremia profiles and the two mathematical functions used to fit these, a binary variable associated with the viremia profile class (uni- or bi-modal) was also fitted as fixed effect; ${\mathbf{a}}_{1}$, ${\mathbf{a}}_{2}$ and ${\mathbf{a}}_{3}$ are vectors of additive genetic effects for each trait, with $${\text{Var}}\left[ {\begin{array}{*{20}c} {{\mathbf{a}}_{1} } \\ {\begin{array}{*{20}c} {{\mathbf{a}}_{2} } \\ {{\mathbf{a}}_{3} } \\ \end{array} } \\ \end{array} } \right] = {\mathbf{G}} \otimes {\mathbf{A}}$$, where ${\mathbf{G}}$ is the genetic variance–covariance matrix and ${\mathbf{A}}$ the pedigree relationship matrix; ${\mathbf{p}}_{1}$, ${\mathbf{p}}_{2}$ and ${\mathbf{p}}_{3}$ are vectors of pen effects nested within a trial for each trait, with $${\text{Var}}\left[ {\begin{array}{*{20}c} {{\mathbf{p}}_{1} } \\ {\begin{array}{*{20}c} {{\mathbf{p}}_{2} } \\ {{\mathbf{p}}_{3} } \\ \end{array} } \\ \end{array} } \right] = {\mathbf{I}} \otimes {\mathbf{K}}$$, where ${\mathbf{I}}$ is the identity matrix and ${\mathbf{K}}$ is the corresponding variance–covariance matrix of pen effects for the different traits; ${\mathbf{l}}_{1}$, ${\mathbf{l}}_{2}$ and ${\mathbf{l}}_{3}$ are the vectors of litter effects for each trait, with $${\text{Var}}\left[ {\begin{array}{*{20}c} {{\mathbf{l}}_{1} } \\ {\begin{array}{*{20}c} {{\mathbf{l}}_{2} } \\ {{\mathbf{l}}_{3} } \\ \end{array} } \\ \end{array} } \right] = {\mathbf{I}} \otimes {\mathbf{L}}$$, with the corresponding variance–covariance matrix ${\mathbf{L}}$; ${\mathbf{e}}_{1}$, ${\mathbf{e}}_{2}$ and ${\mathbf{e}}_{3}$ are the vectors of error terms for each trait, with $${\text{Var}}\left[ {\begin{array}{*{20}c} {{\mathbf{e}}_{1} } \\ {\begin{array}{*{20}c} {{\mathbf{e}}_{2} } \\ {{\mathbf{e}}_{3} } \\ \end{array} } \\ \end{array} } \right] = {\mathbf{I}} \otimes {\mathbf{R}}$$, where ${\mathbf{R}}$ is the variance–covariance matrix for the residual effects for each trait; and ${\mathbf{X}}_{1}$, ${\mathbf{X}}_{2}$ and ${\mathbf{X}}_{3}$, ${\mathbf{Z}}_{1}$, ${\mathbf{Z}}_{2}$ and ${\mathbf{Z}}_{3}$, ${\mathbf{U}}_{1}$, ${\mathbf{U}}_{2}$ and ${\mathbf{U}}_{3}$, and ${\mathbf{M}}_{1}$, ${\mathbf{M}}_{2}$ and ${\mathbf{M}}_{3}$ are the incidence matrices for the fixed, animal, pen and litter effects, respectively. In addition to the trivariate animal model, corresponding bivariate and univariate models were also used to check the robustness of variance components. Since heritability estimates differed between models, heritability estimates were presented from the corresponding univariate models.

#### Step 2: multi-trait models to examine evidence for genetic variation in tolerance—growth associated with low versus high VL

The trivariate model (1) from step 1 does not show how growth changes with respect to viral load, and, therefore, does not account for genetic variance in tolerance. A multi-trait sire model for ADG of progeny with categorized VL was used to assess sire-by-VL interactions to get a first indication of whether sires varied genetically in tolerance to infection. If these genetic correlations are less than 1, this is indicative of sire rank changes when offspring are faced with low and high VL respectively, and provides evidence for genetic variation in tolerance slope. Hence, individuals were sorted according to their VL from 0 to 21 dpi or 0 to 42 dpi, and partitioned into VL groups, where the low and high VL groups (n = 330 each) consisted of individuals with VL values in the lower and upper quartiles, respectively, and the mid-range group consisted of the middle half of the data (n = 660). A trivariate sire model was then fitted to measures of ADG associated with low, mid and high VL from 0 to 21/0 to 42 dpi (ADG_low_, ADG_mid_ and ADG_high_), respectively. The fixed and random effects of this model were identical to those used in model (1), with exception of ${\mathbf{a}}$, which now refers to sire effects on performance and explains one quarter of the additive genetic variance, and of ${\mathbf{e}}$, where residual covariance was fixed at 0, because offspring have only a single record of ADG and therefore the residual covariance does not exist. Furthermore, the pedigree relationship ${\text{A}}$-matrix was replaced with the genomic relationship matrix (${\text{G}}$-matrix) to improve convergence.

#### Step 3: univariate random regression sire models for estimating genetic variance in tolerance

The multi-trait models in the previous steps provide evidence for genetic variation in tolerance but do not yield direct estimates of genetic variance in tolerance. A random regression reaction norm model was applied, whereby the origin of the reaction-norms was centered at the mean viral load values, thus providing only variance component estimates for level (ADG at mean VL) rather than vigor (ADG at zero VL). The following linear random regression sire model (RRM) for ADG on centered values of VL, which will be referred to as the level-slope model (as shown in Fig. 1d), was used:

2 $${\mathbf{y}} = {\mathbf{Xb}} + {\mathbf{X}}_{\text{VL}} {\mathbf{b}}_{{\mathbf{s}}} + {\mathbf{Za}}_{{\mathbf{i}}} + {\mathbf{Z}}_{\text{VL}} {\mathbf{a}}_{{\mathbf{s}}} + {\mathbf{Up}} + {\mathbf{Ml}} + {\mathbf{e}} ,$$

where ${\mathbf{y}}$ is the vector of ADG_21_ or ADG_42_, respectively; ${\mathbf{b}}$ is the vector of fixed effects outlined in model (1), with age and BW_0_ included as additional fixed covariates to account for variation in age and body weight at the start of infection; and ${\mathbf{b}}_{{\mathbf{s}}}$ is the population average tolerance slope; ${\mathbf{a}}_{\varvec{i}}$ and ${\mathbf{a}}_{\varvec{s}}$ are the sire effects on level and on tolerance slope, respectively, assumed to follow a multi-variate normal distribution with mean zero and $${\text{Var}}\left[ {\begin{array}{*{20}c} {{\mathbf{a}}_{{\mathbf{i}}} } \\ {{\mathbf{a}}_{{\mathbf{s}}} } \\ \end{array} } \right] = \frac{1}{4}{\mathbf{G}}_{\text{RN}} \otimes {\mathbf{A}}$$, with $${\mathbf{G}}_{\text{RN}} = \left[ {\begin{array}{*{20}c} {\upsigma_{{{\text{a}}_{\text{i}} }}^{2} } & {\upsigma_{{{\text{a}}_{\text{i}} {\text{a}}_{\text{s}} }} } \\ {\upsigma_{{{\text{a}}_{\text{i}} {\text{a}}_{\text{s}} }} } & {\upsigma_{{{\text{a}}_{\text{s}} }}^{2} } \\ \end{array} } \right]$$, where $\upsigma_{{{\text{a}}_{\text{i}} }}^{2}$ and $\upsigma_{{{\text{a}}_{\text{s}} }}^{2}$ are the variances of ${\text{a}}_{\text{i}}$, and ${\text{a}}_{\text{s}}$, respectively, $\upsigma_{{{\text{a}}_{\text{i}} {\text{a}}_{\text{s}} }}$ is the covariance between sire effects for level and slope; other random effects ${\mathbf{p}}$, ${\mathbf{l}}$, and ${\mathbf{e}}$ were fitted as described in model (1); ${\mathbf{X}}_{\text{VL}}$ and ${\mathbf{Z}}_{\text{VL}}$ are the incidence matrices for population average tolerance slope and those associated with each sire, respectively, consisting of individual VL measures, and ${\mathbf{X}}$ is the incidence matrix for the fixed effects (including VL as fixed covariate) and ${\mathbf{Z}}$ is the incidence matrix for the random sire effect on level (${\text{Z}}$).

To test the significance of sire effects on level and slope and to determine which of the models illustrated in Fig. 1 best described the data, the model fit of the level-slope model (2) was compared with that of hierarchical models: (a) without any additive genetic effects (Fig. 1a), (b) with only sire effects for level (Fig. 1b), and (c) containing only sire effects on slope (Fig. 1c). Significance of each random effect was assessed using the likelihood ratio test (LRT) [31], with the LRT test statistics below assumed to follow a $\chi^{2}$ distribution, with 1 degree of freedom for inclusion of an additional sire effect (e.g. null to level model, including sire effect) and a mixture of 1 and 2 degrees of freedom for additional sire slope effects and covariance (for example, from level to level-slope model) [32, 33].

#### Step 4: random regression model using simulated performance in absence of infection for estimating genetic variance in tolerance

The random regression models fitted in Step 3 generated potential confounding between level and tolerance slope variance estimates i.e. genetic variance in slope was absorbed by genetic variance in level due to the limited distribution of VL around average VL required to estimate the genetic variance in level. To assess whether confounding could be resolved by inclusion of performance measures of non-infected relatives in the statistical models, growth in the absence of infection (ADG_21_^0^ or ADG_42_^0^) was simulated for one hypothetical paternal half-sib for each individual with ADG_21_ and ADG_42_ records, respectively, thus doubling the size of the dataset. Data were simulated assuming a heritability of 0.4 for both ADG_21_^0^ and ADG_42_^0^ [34]. With the expectation that a higher r_g_ between the traits would imply less genetic variance in tolerance, low (0.05), moderate (0.30), strong (0.60) or high (0.90) genetic correlations (r_g_) between ADG_21_^0^ and ADG_21_, or ADG_42_^0^ and ADG_42_, respectively, were simulated (see Additional file 1 for a detailed description of the simulations). Note that no assumptions were made with regards to genetic variance in tolerance. Ten thousand replicates of simulated half-sib records for ADG_21_^0^ and ADG_42_^0^ were generated.

The random regression models (2) were then applied to the extended datasets for each replicate, where the response vector ${\mathbf{y}}$ now comprised either simulated ADG_21_^0^ and measured ADG_21_, or ADG_42_^0^ and ADG_42_. VL was no longer centered at mean VL, but comprised VL equal to zero for the non-infected pigs and VL_21_ or VL_42_ for the infected pigs. The remaining fixed and random effects were identical to those in model (2), except that no fixed effects or random pen or litter effects were fitted for the simulated half-sibs. Thus, by including simulated data of non-infected pigs, model (2) was replaced by an intercept-slope model, with genetic variance estimated for growth in the absence of infection, and for tolerance slope.

As in Step 3, hierarchical models were fitted (a) without any additive genetic effects for intercept or slope (Fig. 1a, null model), and (b) with additive genetic effects for intercept only (Fig. 1b, intercept-only model) and (c) with additive genetic effects for intercept and slope (Fig. 1d, intercept-slope model). The model fit was assessed using the loglikelihood ratio test outlined in Step 3 above. Results were evaluated based on the mean and standard deviation of the estimates over replicates.

## Results

### Step 1: relationship between resistance and performance prior to and post infection

ADG_21_ and ADG_42_ ranged from a weight loss of 40 g/day to a weight gain of 720 and 680 g/day, respectively, with corresponding mean daily weight gains of 280 and 380 g/day (Table 2).

**Table 2:** Summary statistics of resistance and growth traits

Trait	Mean	SD	Min	Max	Number of records
BW_0_ (kg)	7.30	1.39	3.45	12.88	1320
ADG_21_ (kg/day)	0.28	0.12	−0.04	0.72	1319
ADG_42_ (kg/day)	0.38	0.11	−0.04	0.68	1001
VL_21_ (AUC)	115.69	9.37	77.04	153.62	1320
VL_42_ (AUC)	159.90	23.42	88.00	236.35	1001

Body weight at 0 dpi (BW_0_), average daily gain and viral load from 0 to 21 and 0 to 42 dpi (ADG_21_, ADG_42_, VL_21_ and VL_42_), respectively

AUC is the area under the curve for the log-transformed estimates for viral load in blood as measured by RT-PCR

**Fig. 2:**
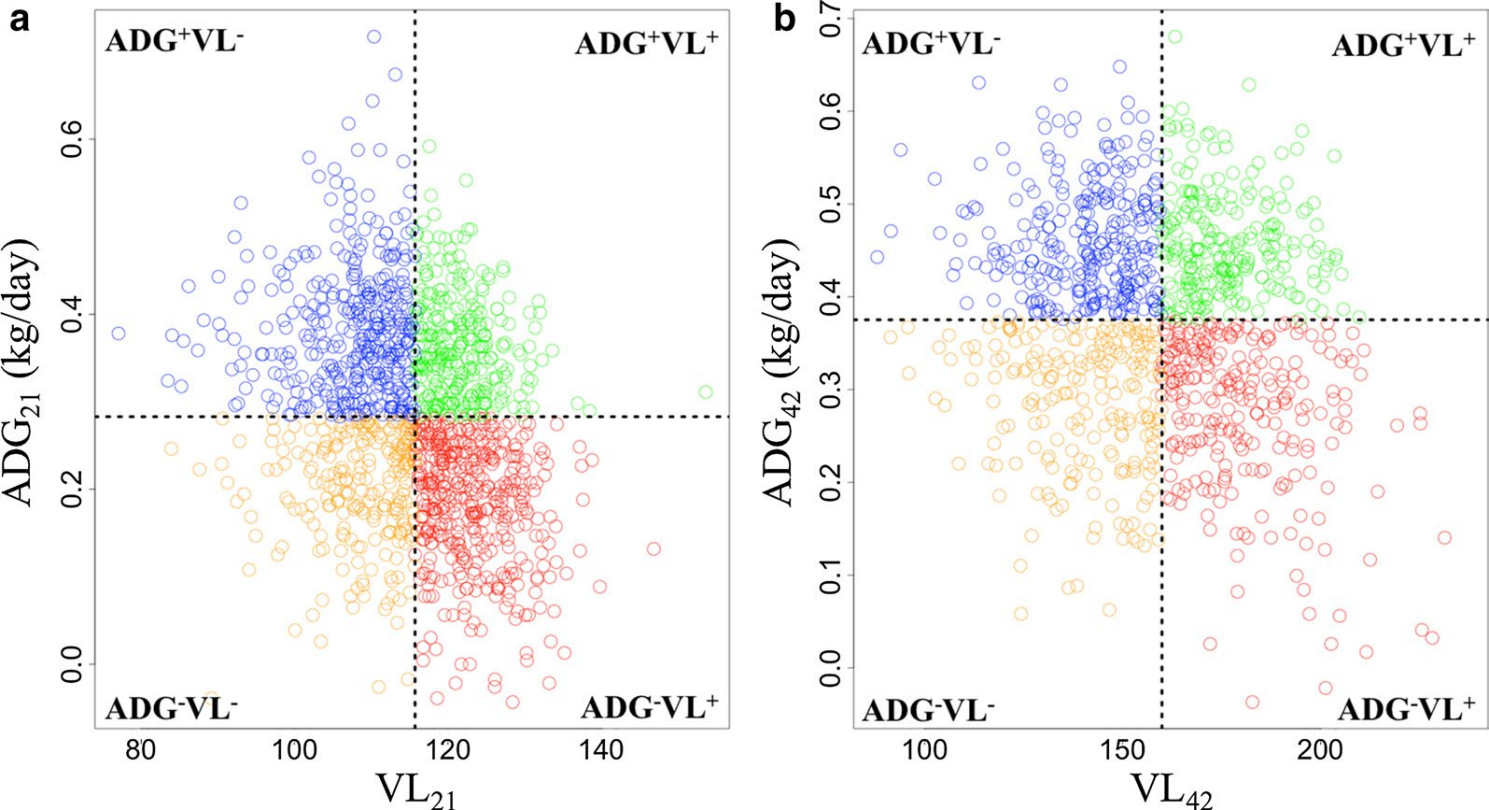
Scatter plots of data for ADG and VL from **a** 0 to 21 and **b** 0 to 42 dpi. ADG and VL from 0 to 21 and 0 to 42 dpi (n = 1320 and 1001, respectively) were distributed into one of four quadrants according to their growth and VL after infection with PRRS virus (n = 330 and 250 in each quadrant for 0 to 21 and 0 to 42 dpi, respectively). The quadrants (ADG^+^VL^−^ blue, ADG^+^VL^+^ green, ADG^−^VL^−^ orange, and ADG^−^VL^+^ red) refer to high growth rate and high resistance (low VL), high growth rate and low resistance (high VL), low growth rate and high resistance and low growth rate and high low, respectively. Quadrants were centered at mean VL and at mean ADG

Figure 2 depicts the distributions of growth and VL for the two observation periods between 0 to 21 dpi and 0 to 42 dpi. The wide distribution of individuals with above average growth rate in spite of high VL (ADG^+^VL^+^), and with low growth rate in spite of low VL (ADG^−^VL^−^) may be indicative of phenotypic variation in tolerance.

Growth rate under infection and resistance were moderately heritable and had large standard errors (Table 3). Heritability estimates were similar for the two time periods considered.

**Table 3:** Estimates of heritability and correlations between resistance and growth traits

Trait	Trait				
	BW_0_	ADG_21_	ADG_42_	VL_21_	VL_42_
BW_0_	*0.11 (0.10)*	0.35 (0.03)	0.40 (0.03)	−0.21 (0.07)	−0.20 (0.03)
ADG_21_	0.48 (0.30)	0.*29 (0.11)*	0.80 (0.01)	−0.29 (0.03)	–
ADG_42_	0.24 (0.45)	1.00 (0.04)	*0.34 (0.14)*	−0.33 (0.03)	−0.36 (0.03)
VL_21_	−0.33 (0.45)	−0.53 (0.27)	−0.64 (0.26)	*0.19 (0.11)*	0.80 (0.01)
VL_42_	−0.54 (0.37)	–	−0.82 (0.16)	0.79 (0.14)	*0.18 (0.10)*

Heritability estimates (diagonal) and phenotypic (upper triangle) and genetic correlations (lower triangle) with standard errors (SE) from the trivariate animal model for body weight at 0 dpi (BW_0_), average daily gain and viral load from 0 to 21 and 0 to 42 dpi (ADG_21_, ADG_42_, VL_21_ and VL_42_), respectively

Correlations between ADG_21_ and VL_42_ were not calculated, since VL is expected to impact ADG and not the other way around

Although standard errors were high, genetic correlations between VL and growth under infection were statistically significantly different from 1 (*p* < 0.001, based on the LRT that compares models with and without genetic correlations fixed to 1), indicating that not all genetic variation of growth under infection was explained by genetic differences in resistance (inverse of VL) (Table 3). Furthermore, genetic correlations between growth under infection and BW_0_ were also significantly different from 1, implying that growth prior to and post infection were not under identical genetic regulation. Genetic correlations between growth under infection and VL were moderate to strong and negative whereas genetic correlations between growth under infection and BW_0_ were moderately positive. Phenotypic correlations were of the same sign but generally weaker than the genetic correlations (Table 3). Phenotypically and genetically, these results indicate that pigs with greater resistance tend to grow faster.

### Step 2: multi-trait models to examine evidence for genetic variation in tolerance

Trivariate models for growth at low, mid and high VL failed to converge for both time periods of infection. Using bivariate models for the upper and lower quartiles for VL, high genetic correlations of 0.94 (0.18) and 0.91 (0.13) between growth associated with low to high VL were identified for ADG_21_ and ADG_42_, respectively. Genetic correlations significantly less than 1 would imply that growth rates associated with different degrees of infection severity, as indicated by low versus high VL, are genetically distinct traits and would thus be indicative of genetic variation in tolerance (Fig. 1). Genetic correlations close to 1 indicate limited reranking among sires between high and low levels of VL and, thus limited genetic variance in tolerance. Furthermore, there was no significant difference between genetic variances of ADG associated with low and high VL, for either the 0 to 21 and 0 to 42 day period (where genetic variances for ADG associated with low and high VL were 2.10E^−03^ (1.22E^−03^) and 4.56E^−03^ (1.81E^03^) for 0 to 21 dpi, and 3.46E^−03^ (1.24E^−03^) and 6.89E^−03^ (2.18E^−03^) for 0 to 42 dpi, respectively). Referring to the expectations outlined in Fig. 1, the results of this multi-trait model imply that random regression models of Step 3 with the same tolerance slope for each sire would provide a better fit to the data than models with different slopes for each sire (Fig. 1c, d).

### Step 3: estimation of genetic variance in tolerance using univariate random regression models

Univariate random regression models without genetic effects, but including VL as a fixed linear (and higher order polynomial) covariate were used to test the average association between growth and VL (null model in Table 4). These identified a statistically significant linear association between growth and VL (*p* < 0.0001), with a population average tolerance slope estimate of −2.78E^−03^ (3.32E^−04^) and −1.28E^−03^ (1.51E^−04^) kg/day per unit of VL increase for ADG_21_ regressed on VL_21_ and ADG_42_ on VL_42_, respectively. This corresponds to an average growth rate difference of 213 and 190 g/day between pigs with the lowest and highest observed VL for the 21- and 42-day observation period, respectively, or differences in body weight of 4.5 and 8.0 kg over the 21- and 42-day observation periods, respectively. Similarly, body weight prior to infection had a significant association with ADG post infection (BW_0_ *p* < 0.0001), with a positive regression coefficient of 0.025 (0.002) at 21 dpi and of 0.029 (0.003) at 42 dpi.

**Table 4:** Variance components for ADG (kg/d) from 0 to 21 dpi and 0 to 42 dpi

ADG period (dpi)	Null model	Level-only model	Slope-only model	Level-slope model
	Estimate (SE)	Estimate (SE)	Estimate (SE)	Estimate (SE)
0 to 21				
Level		2.01E−03 (7.68E−04)		2.01E−03 (7.68E−04)
Covariance				2.21E−13 (1.04E−14)
Slope			4.37E−06 (2.06E−07)	1.00E−10 (4.71E−12)
Pen (trial)	4.12E−04 (1.45E−04)	3.97E−04 (1.42E−04)	4.12E−04 (1.45E−04)	3.97E−04 (1.42E−04)
Litter	9.25E−04 (2.26E−04)	4.72E−04 (2.04E−04)	9.25E−04 (2.26E−04)	4.72E−04 (2.04E−04)
Residual	6.18E−03 (2.91E−04)	6.18E−03 (2.91E−04)	6.18E−03 (2.91E−04)	6.18E−03 (2.91E−04)
LogLikelihood	2482.98	2495.03	2482.98	2495.2
0 to 42				
Level		2.32E−03 (1.02E−03)		2.33E−03 (1.03E−03)
Covariance				−3.95E−15 (2.04E−16)
Slope			8.60E−08 (1.47E−07)	3.41E−07 (5.94E−07)
Pen (trial)	2.43E−04 (1.30E−04)	2.82E−04 (1.36E−04)	2.39E−04 (1.29E−04)	2.78E−04 (1.35E−04)
Litter	1.76E−03 (3.27E−04)	1.23E−03 (2.98E−04)	1.75E−03 (3.27E−04)	1.22E−03 (2.98E−04)
Residual	5.39E−03 (3.03E−04)	5.33E−03 (2.99E−04)	5.36E−03 (3.05E−04)	5.30E−03 (3.02E−04)
LogLikelihood	1889.55	1911.18	1899.72	1911.35

Variance components estimated from random regression models: null model, containing no genetic effect; level-only model, containing only the overall sire effect on growth under infection; slope-only model, containing only sire effect on the slope of the regression line of growth over VL; and level-slope model, containing sire effects on level and slope, respectively

All other fixed effects/covariates and random effects were identical between models

Results for ADG_42_ on VL_21_ were similar to those for ADG_42_ on VL_42_ and are therefore not shown

The log-likelihood of the model improved significantly when genetic effects (random sire effects) were included in the model (level model) (*p* < 0.0001) (Table 4). This indicates significant genetic variance in growth performance of pigs infected with PRRSV. However, including sire effects of slope only (Fig. 1c) did not improve model fit over the null model (*p* > 0.60) and resulted in negligibly small slope variance estimates.

Models with sire effects on both level and slope, as well as a genetic covariance between them, yielded a significantly better model fit than the null model (*p* < 0.0001). However, the level-slope model did not provide a significantly better fit than the level-only model for either 0 to 21 and 42 dpi (*p* = 1.00 and 0.66, respectively) (Table 4).

All four models provided similar estimates of variance components for non-genetic random effects (Table 4). Estimates of the sire variance in level were very similar between the level-only model and the level-slope model and very low, whereas estimates for sire variance in tolerance slope differed slightly between the slope-only and the level-slope model (Table 4). The fact that addition of the slope did not affect the variance estimate for level suggests potential confounding of level and slope (see statistical considerations). The estimate of the covariance between level and slope was close to zero, and constrained at the boundary for both time periods, indicating numerical difficulties in accurately estimating these variance components. However, shifting the covariate VL to ensure a zero covariance between the new level and slope has no effect on the model likelihoods, suggesting that the results are robust.

In conclusion, the random regression models did not allow estimation of genetic variance in tolerance of pigs to PRRSV infection. Based on a statistical model fit alone, the level-only model accounting for genetic variance in growth rate at mean VL only constitutes a more appropriate model to describe genetic variation in growth response of infected pigs than the level-slope model accounting for genetic variance in both, growth rate at mean VL and tolerance. However, as outlined in more detail in the “statistical considerations” section below, it cannot be excluded that any genetic variance in tolerance that may exist is absorbed in the genetic variance for level because of the confounding between level and slope.

### Step 4: random regression models including simulated performance in absence of infection for estimating genetic variance in tolerance

Models with genetic effects on both intercept and slope, as well as with a genetic covariance between them, consistently yielded a significantly better model fit than the null model (*p* < 0.0001 for both 0 to 21 and 0 to 42 dpi), regardless of the simulated genetic correlation between ADG_21_^0^ and ADG_21_ or ADG_42_^0^ and ADG_42_. However, the intercept-slope model consistently provided a significantly superior fit over the intercept-only model only when the simulated genetic correlation between growth in absence of infection and growth under infection was low to moderate (Table 5). Generally, the ability to identify genetic variance in tolerance decreased with an increase in the simulated genetic correlation, as indicated by reduced improvement in log-likelihoods and a lower proportion of replicates with significant genetic variation in tolerance slope (*p* < 0.05) (Table 5). Somewhat surprisingly, for the 0 to 21 dpi observation period, the majority of replicates indicated significant genetic variation in tolerance, even for strong genetic correlations between ADG_21_^0^ and ADG_21_ (Table 5). In contrast, only low to moderate genetic correlations between ADG_42_^0^ and ADG_42_ resulted in significant genetic variance in tolerance for the majority of replicates for the 42 day observation period (Table 5).

**Table 5:** Effect of the genetic correlation (r_g_) between simulated ADG in the absence of infection and observed ADG under infection on evidence for genetic variance in tolerance

ADG period (dpi)	r_g_	ΔLogLikelihood	*p* value	Proportion with significant genetic variance for tolerance (*p* < 0.05)
0 to 21	0.05	10.96 (4.19)	0.000	1.00
	0.30	6.18 (3.39)	0.005	0.98
	0.60	2.32 (1.49)	0.041	0.76
	0.90	1.00 (2.12)	0.067	0.55
0 to 42	0.05	8.67 (4.40)	0.003	0.99
	0.30	4.34 (3.16)	0.023	0.87
	0.60	1.31 (1.56)	0.107	0.41
	0.90	−0.80 (2.43)	0.187	0.06

Effect of the genetic correlation (r_g_) of simulated ADG_21_^0^ with ADG_21_ and ADG_42_^0^ with ADG_42_ on the average change in log-likelihood of the intercept-slope model over the intercept-only model (∆LogLikelihood), the average p-value of log likelihood improvement, provided by a log-likelihood ratio test, and the proportion of the 10,000 replicates with significant genetic variance in tolerance (i.e. *p* value of LRT was <0.05)

SD over 10,000 replicates are shown in brackets

Table 6 shows that random regression sire models when including records from both non-infected and infected siblings can generate robust genetic variance estimates for both intercept and slope. As expected, genetic variance estimates for tolerance slope tended to decrease with increasing genetic correlations between ADG_21_^0^ and ADG_21_ or ADG_42_^0^ and ADG_42_, whereas the genetic variance estimates in the intercept tended to increase (see Additional file 1). Genetic correlations beween ADG in absence of infection and ADG under infection also affected the estimated genetic correlations between intercept and tolerance slope. Low (simulated) genetic correlations between ADG_21_^0^ and ADG_21_ or ADG_42_^0^ and ADG_42_, respectively, led to negative genetic correlations between performance in the absence of infection and tolerance, whereas strong positive genetic correlations between the growth traits suggested that pigs with greater genetic growth in the absence of infection were also genetically more tolerant to infection.

**Table 6:** Variance components of intercept, slope and covariances from random regression models

ADG period (dpi)	r_g_	Intercept-only model	Intercept–slope model		
		Intercept	Intercept	Covariance	Slope
0 to 21	0.05	7.65E−04 (2.24E−04)	9.93E−04 (2.98E−04)	−7.57E−06 (3.97E−06)	2.24E−07 (5.80E−08)
	0.3	9.20E−04 (2.53E−04)	9.95E−04 (2.99E−04)	−3.03E−06 (3.27E−06)	1.44E−07 (5.09E−08)
	0.6	1.13E−03 (2.71E−04)	1.03E−03 (2.84E−04)	1.54E−06 (2.13E−06)	5.54E−08 (3.24E−08)
	0.9	1.34E−03 (2.31E−04)	1.19E−03 (1.95E−04)	3.35E−06 (1.06E−06)	1.25E−08 (6.33E−09)
0 to 42	0.05	9.20E−04 (2.83E−04)	1.10E−03 (3.47E−04)	−5.80E−06 (3.59E−06)	1.18E−07 (3.87E−08)
	0.3	1.09E−03 (3.05E−04)	1.12E−03 (3.47E−04)	−1.90E−06 (2.85E−06)	6.85E−08 (3.23E−08)
	0.6	1.28E−03 (3.15E−04)	1.14E−03 (3.19E−04)	−5.57E−07 (1.75E−06)	2.37E−08 (1.65E−08)
	0.9	1.47E−03 (2.57E−04)	1.21E−03 (4.10E−04)	1.95E−06 (1.21E−06)	1.09E−08 (7.13E−09)

Variance components estimated from random regression models based on simulated ADG_21_^0^ and measured ADG_21_ (kg/d) or ADG_42_^0^ and ADG_42_

Fitted models were the intercept-only model, containing only the overall sire effect on intercept; and the intercept-slope model, containing sire effect on intercept and slope for ADG_21_ or ADG_42_, respectively

All other fixed effects/covariates and random effects were identical between models

SE (in brackets) were calculated as the SD over 10,000 replicates

r_g_ is the simulated genetic correlation between ADG_21_^0^ or ADG_42_^0^ and ADG_21_ or ADG_42_

## Discussion

### Summary of findings

Performance of an infected individual is likely to depend on its ability to restrict pathogen load (resistance) and its ability to limit the impact of infection (tolerance). The extensive PHGC dataset has identified substantial genetic variation in resistance of growing pigs to PRRS and led to the discovery of a major quantitative trait locus associated with both resistance and growth of pigs under infection [10, 12, 13, 20]. Surprisingly, the dataset provided little evidence that pigs also vary genetically in tolerance to this virus. However, the simulations revealed that genetic variation in tolerance to PRRS may exist, depending on the performance in the absence of infection (vigor). Furthermore, this analysis raised numerous statistical difficulties associated with genetic improvement of host tolerance, which could be overcome by including measures of performance of infected and non-infected relatives in the analysis.

Focusing on data from infected pigs alone, genetic correlations between body weight prior to infection, resistance (inverse of VL) and growth under infection were found to be moderately strong and positive, in line with previous studies [10, 20]. This indicates that heavier individuals prior to infection counteract an increase in pathogen load, and thus tend to have lower VL, and therefore lower infection-induced reductions in growth rate. Genetic correlations between VL and growth were strongly negative, implying that animals that were genetically more resistant also tended to grow faster under infection. However, correlations were significantly different from 1, indicating that genetic variation in growth of PRRSV infected pigs is not fully explained by heterogeneity in growth prior to infection and resistance. Therefore, genetic variation in tolerance may also play a part in host response to PRRSV infection. However, the multi-trait model provided little evidence of genetic variation in tolerance. This was further supported by the random regression models. These showed that, although growth rate declined, on average, linearly with increasing VL, there was no statistically significant difference in tolerance between the sires of the infected piglets.

However, closer inspection of the underlying data structure raised suspicion that genetic variance in the reaction norm level absorbed genetic variance in tolerance due to confounding between level and slope in these data (see Statistical considerations below). To disentangle the genetic variance in reaction-norm intercepts (i.e. growth rate in the absence of infection) and slopes (i.e. tolerance), the experimental data were augmented with simulated growth rates of non-infected relatives. Thus, the resulting data structure mimicked that of ‘sib challenge tests’ that are common practice in aquaculture and other livestock species [35–37]. The simulations demonstrated that inclusion of these additional data in the random regression models resolved the confounding between level and slope and resulted in more reliable genetic parameter estimates for tolerance. Most importantly, the simulations revealed that it would be wrong to conclude that pigs in this study lacked substantial genetic variation in tolerance to PRRS, as was suggested by the models based on the collected data alone. As demonstrated by the simulations, genetic variance estimates for tolerance strongly depend on the genetic correlations between growth in the absence of and growth under infection. Low to moderately strong genetic correlations between these two traits implied significant genetic variance in tolerance of the pigs in this study. Interestingly, estimated genetic correlations between body weight of pigs prior to infection and growth under infection were moderately strong. Thus, if body weight prior to infection was a reliable predictor for growth rate in the absence of infection, evidence for genetic variance in tolerance would emerge directly from the data.

### Statistical considerations

Here, the conventional reaction-norm approach was adopted to model genetic variation in tolerance to infections [2, 38]. Using both simulated and real data, we demonstrated that random regression models embedded in the mixed model machinery are a powerful tool to estimate genetic variance in tolerance for outbred populations if the data structure is appropriate [15, 16, 18]. Random regression models are also known to be highly sensitive to the underlying data structure and prone to generate inaccurate variance estimates for slope, in particular, if sample size is limited or information on relatedness is poor, as was the case for the data in this study [15, 17, 30]. To prevent bias in the slope variance estimates [15, 30], only sires that had more than 10 offspring were included in this study. However, the associated reduction of the data to records from only 54 mostly unrelated sires may have caused a trade-off between reducing bias and reducing statistical power, as indicated by lower heritabilities for ADG and VL than found in previous analyses on the same data [10, 20]. Furthermore, to alleviate the potential impact of limited information of relatedness (as only sires and dams were known for the majority of pigs), the analyses were repeated including the genomic relationship matrix rather than the pedigree relationship matrix, which is not able to capture the difference between siblings due to Mendelian sampling. However, this had a negligible impact on the variance estimates and on the log-likelihoods of the reaction-norm models (results not shown).

As is common practice for quantitative genetics models using REML, the likelihood ratio test (LRT) was used to test the significance of random effects such as the sire tolerance slope estimates, and whether genetic correlations differed significantly from 1 [39]. For variance and co-variance components constrained to the positive parameter space, the conventional LRT that assumes the test-statistics to follow a Chi square distribution with degrees of freedom equal to the number of additional parameters to be estimated in the more complex model has been described to be overly conservative [23]. For this reason the widely used adjustment of Stram and Lee [32] based on mixture distributions was applied. However, in this proposed adjustment, individual subjects (in this case sires) were assumed independent. Due to lack of detailed pedigree information in the present study, the majority of sires were indeed assumed unrelated, with the exception of sires from trials 1 to 3. Repeating the analysis with the assumption that all sires were unrelated provided almost identical model results to those reported here. Thus, we believe that the LRT is a valid method for testing the null hypotheses of zero genetic variance in tolerance and genetic correlations equal to 0 or 1 in this study. Nevertheless, sires and sire by VL interactions were also fitted as fixed effects in the statistical models of Step 3. In accordance with the results of modelling sires as random effect, there were no significant differences between the tolerance slopes associated with different sires according to the Wald test (*p* = 0.981 and 0.081 for the 0 to 21 and 0 to 42 dpi time periods, respectively).

Perhaps most importantly, reaction-norms require considerable variation in the independent variable to generate unbiased tolerance slope estimates [1]. However, this study, in line with other infection challenge experiments, used an identical infection route, pathogen strain and dose for all individuals. Consequently, it provided a relatively narrow value range for pathogen load (VL_42_ values ranged between 88 and 236 AUC in our study), with no values close to 0. To better accommodate the distribution of the data in the models, the VL was centered at the mean VL value, in line with common practice in the animal breeding literature [17, 27, 30]. However, the relatively narrow range of the VL of offspring, combined with the relatively small numbers of offspring for some sires, may have hampered the ability of these models to disentangle sire effects on level and slope. This confounding is likely further aggravated by genetic variation in resistance to PRRS, which implies that VL is not homogeneously distributed among sires, with more resistant sires predominantly having progeny with low VL, and less resistant sires predominantly progeny with high VL.

Considering all these effects combined, the weak evidence for significant genetic variation in tolerance to PRRS from the random regression models in this study may simply reflect a lack of statistical power to disentangle sire effects on regression slope and level. The complementary simulation studies presented here, which assumed that additional performance measures of related uninfected individuals were available, demonstrated one way of increasing statistical power. Similarly, it might be possible to increase statistical power by harnessing information from repeated measures of growth and pathogen load for each individual over the course of infection in the statistical models. By increasing the range of distribution of VL for each individual, a more robust slope may be fitted through the centre of the data, alluding to an “overall” picture of tolerance across multiple time-points in infection.

### Implications for genetic improvement of tolerance of pigs to PRRS and other diseases

Genetic improvement of tolerance may have several advantages over improving resistance. Firstly, host resistance limits pathogen replication within the host and, as a consequence, selection for host resistance may impose selection advantages on pathogen strains that can overcome host resistance mechanisms and eventually result in a loss of selection advantage of the host [40, 41]. Given the high mutation rate of RNA viruses such as PRRSV [42], this is a potential pitfall for a long-term breeding strategy focused on resistance. It has been proposed that, theoretically, tolerance might not impose such selection pressure on the pathogen [40].

Secondly, it has been suggested that improving host tolerance may offer cross-protection against other strains of the virus, or other prevalent infectious agents, as tolerance mechanisms primarily target host-intrinsic damage prevention or repair mechanisms, compared to resistance mechanisms, which interfere directly with the pathogen life-cycle [2, 5, 43]. This is particularly relevant for PRRS, which is often associated with co-infection with other respiratory viruses, such as PCV2 or the influenza virus, which can mimic the respiratory clinical signs associated with PRRS [44]. Furthermore, in a globalized animal breeding market, where PRRS is endemic and highly prevalent in farms, (estimated at 60 to 80% in the U.S, and up to 79% in mainland Europe), and where environmental conditions are difficult to improve, eradication of the virus has proven to be challenging [45–47]. Selective breeding for tolerance is considered desirable when pathogen prevalence is high, when pathogen elimination has proven difficult and when pathogens can evolve rapidly to evade control measures that aim at interfering with the pathogen life-cycle [48]. All these cases apply to PRRS. Therefore, improvement of tolerance of pigs to this ubiquitous virus may constitute a viable alternative to eradication programs, since it would allow pigs to maintain homeostasis despite infection [44]. However, tolerance would result in continued presence of the virus which could rebound and result in further pathogenesis in the host and threats to the herd. Thus, distinction between resistance and tolerance in genetic improvement programs is imperative if they have different effects on pathogen prevalence and evolution, as implied by theory [40, 49].

Obtaining reliable tolerance estimates from natural disease outbreaks is extremely difficult due to the myriad of confounding factors (e.g. difference in exposure and onset of infection, differences in the individual immune status, co-infections), which can severely bias tolerance estimates and mask the underlying genetic signal [16, 18]. For this reason, empirical evidence for genetic variation in host tolerance to infections stems primarily from challenge experiments in inbred lines of model species [2, 50, 51]. The PHGC challenge data constitute a unique data source for investigating the genetic basis and relative importance of host resistance and tolerance in outbred pigs’ responses to virus infections, since it provides the required measures of both pathogen load and performance for large sample sizes, without the confounding factors inherent to field data. However, the analyses of these data demonstrated that the limited data range produced in challenge experiments, together with other factors that affect the distribution of the data, such as genetic variance in host resistance, can easily blur the tolerance signal in multi-trait and reaction-norm models, and highlight the importance of performance records of non-infected relatives for obtaining accurate tolerance estimates.

Collecting equivalent performance records of non-infected relatives of the challenged individuals would be extremely valuable to establish the relationship between tolerance and performance in the absence of infection, and identify shared or distinct genomic regions associated with these traits. A strong genetic correlation between these traits would imply that one could select for high performance at the nucleus to improve tolerance and performance in the more infectious commercial farms. In the current pig breeding structure, a direct data pipeline of performance measures between pigs in commercial farms experiencing disease outbreaks and those of related selection candidates in the almost pathogen free nucleus may be useful. Obtaining unbiased and comparable measures of within-host pathogen load from natural disease outbreaks constitutes the main challenge for producing reliable tolerance estimates from natural disease outbreaks [16]. A practically more feasible approach is to estimate genetic correlations between performance in clean and infectious environments and to include performance during disease outbreaks in the selection criterion [52, 53], although this approach does not allow distinction between resistance and tolerance.

Based on resource-allocation theory and earlier findings, resistance and tolerance are conventionally considered as alternative host defense mechanisms to infections, leading to the notion of a trade-off between improving resistance and tolerance. Indeed, a companion genome-wide association study on the same PHGC data found different regions that were associated with tolerance and with resistance [54]. Emerging evidence from different studies suggests that both resistance and tolerance mechanisms may be required for effective host protection to infection and that the optimal host response to infection likely depends on a carefully timed interaction between pathogen elimination (i.e. resistance) mechanisms and host mechanisms that promote tissue damage control and increase disease tolerance [51, 55]. The aforementioned companion study identified several overlapping genomic regions associated with resistance and tolerance of pigs to PRRS and found that the WUR10000125 SNP, previously associated to confer greater resistance to PRRS (lower VL_21_), also confers greater tolerance. Valuable insights about these interactions could be harnessed from the available longitudinal measures of pathogen burden and growth, e.g. by following the infection trajectories of individuals and target entire trajectories rather than resistance or tolerance for genetic improvement [51, 56].

In order to target both resistance and tolerance in a sustainable breeding program, the epidemiological and evolutionary consequences of genetic selection in either or both traits combined must be studied in more detail. In particular, it needs to be determined whether evolutionary theory predicts a lower risk of pathogen evolution from selection for improved host tolerance rather than resistance hold in the case of PRRS; and to what extent genetically more resistant or tolerant pigs are also less infectious [3, 57, 58]. It is probable that control of PRRS and other infectious diseases by genetic selection is a “balancing act” [9], which involves mechanisms associated with resistance and tolerance to provide the fittest pigs.

## Conclusions

Using evidence from the available data alone suggests that growing piglets differ genetically in resistance but does not explicitly show evidence for genetic differences in tolerance to PRRSV infection. However, statistical constraints may have masked genetic variation in tolerance. Currently, unknown genetic correlations between performance under and in absence of PRRSV infection could reveal significant genetic variance in tolerance. Future studies are warranted to validate the results in this study for infections with the same and different strains of the PRRS virus, including vaccine strains. This study shows that genetics of tolerance is more difficult to analyze than genetics of resistance, and is therefore more difficult to target in genetic improvement.

## Additional file

**Additional file 1.** Simulating ADG in absence of infection. The additional file provides a detailed methodology on how performance in absence of infection was simulated.

## References

[1] Råberg L, Graham AL, Read AF, Raberg L, Graham AL, Read AF (2009). Decomposing health: tolerance and resistance to parasites in animals. Philos Trans R Soc B Biol Sci.

[2] Råberg L, Sim D, Read AF, Raberg L, Sim D, Read AF (2007). Disentangling genetic variation for resistance and tolerance to infectious diseases in animals. Science.

[3] Vale PF, McNally L, Doeschl-Wilson A, King KC, Popat R, Domingo-Sananes MR (2016). Beyond killing. Evol Med Public Health.

[4] Doeschl-Wilson AB, Kyriazakis I (2012). Should we aim for genetic improvement in host resistance or tolerance to infectious pathogens?. Front Genet.

[5] Medzhitov R, Schneider DS, Soares MP (2012). Disease tolerance as a defense strategy. Science.

[6] Doeschl-Wilson AB, Kyriazakis I, Vincent A, Rothschild MF, Thacker E, Galina-Pantoja L (2009). Clinical and pathological responses of pigs from two genetically diverse commercial lines to porcine reproductive and respiratory syndrome virus infection. J Anim Sci.

[7] Holtkamp DJ (2013). Assessment of the economic impact of porcine reproductive and respiratory syndrome virus on United States pork producers. J Swine Health Prod.

[8] Kimman TG, Cornelissen LA, Moormann RJ, Rebel JMJ, Stockhofe-Zurwieden N (2009). Challenges for porcine reproductive and respiratory syndrome virus (PRRSV) vaccinology. Vaccine.

[9] Lunney JK, Chen H (2010). Genetic control of host resistance to porcine reproductive and respiratory syndrome virus (PRRSV) infection. Virus Res.

[10] Hess AS, Islam ZZ, Hess MK, Rowland RRRR, Lunney JJK, Doeschl-Wilson AA (2016). Comparison of host genetic factors influencing pig response to infection with two North American isolates of porcine reproductive and respiratory syndrome virus. Genet Sel Evol.

[11] Lewis CRG, Ait-Ali T, Clapperton M, Archibald AL, Bishop S (2007). Genetic perspectives on host responses to porcine reproductive and respiratory syndrome (PRRS). Viral Immunol.

[12] Boddicker NJ, Garrick DJ, Rowland RRR, Lunney JK, Reecy JM, Dekkers JCM (2013). Validation and further characterization of a major quantitative trait locus associated with host response to experimental infection with porcine reproductive and respiratory syndrome virus. Anim Genet.

[13] Boddicker NJ. The genetic basis of host response to experimental infection with the porcine reproductive and respiratory syndrome virus in pigs [dissertation]. 2013;198

[14] Simms EL, Triplett J (1994). Costs and benefits of plant-responses to disease—resistance and tolerance. Evolution.

[15] Kause A (2011). Genetic analysis of tolerance to infections using random regressions: a simulation study. Genet Res.

[16] Doeschl-Wilson AB, Villanueva B, Kyriazakis I (2012). The first step toward genetic selection for host tolerance to infectious pathogens: obtaining the tolerance phenotype through group estimates. Front Genet.

[17] Knap PW, Su G (2008). Genotype by environment interaction for litter size in pigs as quantified by reaction norms analysis. Animal.

[18] Hayward AD, Nussey DH, Wilson AJ, Berenos C, Pilkington JG, Watt KA, et al. Natural selection on individual variation in tolerance of gastrointestinal nematode infection. PLoS Biol. 2014;12(7):e1001917. 10.1371/journal.pbio.1001917 PMC4114752 25072883

[19] Truong HM, Lu Z, Kutish GF, Galeota J, Osorio FA, Pattnaik AK (2004). A highly pathogenic porcine reproductive and respiratory syndrome virus generated from an infectious cDNA clone retains the in vivo virulence and transmissibility properties of the parental virus. Virology.

[20] Boddicker N, Waide EH, Rowland RRR, Lunney JK, Garrick DJ, Reecy JM (2012). Evidence for a major QTL associated with host response to porcine reproductive and respiratory syndrome virus challenge. J Anim Sci.

[21] Islam ZU, Bishop SC, Savill NJ, Rowland RRR, Lunney JK, Trible B, et al. Quantitative analysis of porcine reproductive and respiratory syndrome (PRRS) viremia profiles from experimental infection: a statistical modelling approach. PLoS One. 2013;8(12):e83567. 10.1371/journal.pone.0083567 PMC3866253 24358295

[22] Ramos AM, Crooijmans RPMA, Affara NA, Amaral AJ, Archibald AL, Beever JE, et al. Design of a high density SNP genotyping assay in the pig using SNPs identified and characterized by next generation sequencing technology. PLoS One. 2009;4:e6524. 10.1371/journal.pone.0006524 PMC2716536 19654876

[23] Gilmour AR, Gogel BJ, Cullis BR, Thompson R. ASReml User Guide Release 3.0. Hemel Hempstead: VSN International Ltd; 2009.

[24] VanRaden PM (2008). Efficient methods to compute genomic predictions. J Dairy Sci.

[25] Stowe KA, Marquis RJ, Hochwender CG, Simms EL (2000). The evolutionary ecology of tolerance to consumer damage. Annu Rev Ecol Syst.

[26] Schaeffer LR (2004). Application of random regression models in animal breeding. Livest Prod Sci.

[27] Kolmodin R, Bijma P (2004). Response to mass selection when the genotype by environment interaction is modelled as a linear reaction norm. Genet Sel Evol.

[28] Van Tienderen PH, Koelewijn HP (1994). Selection on reaction norms, genetic correlations and constraints. Genet Res.

[29] Strandberg E. Analysis of genotype by environment interaction using random regression models. In: Proceedings of 8th world congress on genetics applied to livestock production, Belo Horizonte, Minas Gerais, Brazil, 13–18 August 2006; 2006. p. 25–05.

[30] Calus MPL, Bijma P, Veerkamp RF (2004). Effects of data structure on the estimation of covariance functions to describe genotype by environment interactions in a reaction norm model. Genet Sel Evol.

[31] Lynch M, Walsh B (1998). Genetics and analysis of quantitative traits.

[32] Stram DO, Lee JW (1994). Variance components testing in the longitudinal mixed effects model. Biometrics.

[33] Visscher PM, Medland SE, Ferreira MAR, Morley KI, Zhu G, Cornes BK (2006). Assumption-free estimation of heritability from genome-wide identity-by-descent sharing between full siblings. PLoS Genet.

[34] Chen P. Genetic improvement of lean growth rate and reproductive traits in pigs. http://lib.dr.iastate.edu/rtd.

[35] Ødegård J, Meuwissen TH, Meuwissen T, Hayes B, Goddard M, Luan T (2014). Identity-by-descent genomic selection using selective and sparse genotyping. Genet Sel Evol.

[36] Kause A, van Dalen S, Bovenhuis H (2012). Genetics of ascites resistance and tolerance in chicken: a random regression approach. G3 (Bethesda).

[37] de Greef KH, Janss LL, Vereijken AL, Pit R, Gerritsen CL (2001). Disease-induced variability of genetic correlations: ascites in broilers as a case study. J Anim Sci.

[38] Simms EL (2000). Defining tolerance as a norm of reaction. Evol Ecol.

[39] Wilson AJ, Réale D, Clements MN, Morrissey MM, Postma E, Walling CA (2010). An ecologist’s guide to the animal model. J Anim Ecol.

[40] Roy BA, Kirchner JW (2000). Evolutionary dynamics of pathogen resistance and tolerance. Evolution.

[41] Restif O, Koella JC (2004). Concurrent evolution of resistance and tolerance to pathogens. Am Nat.

[42] Drake JW, Holland JJ (1999). Mutation rates among RNA viruses. Proc Natl Acad Sci USA.

[43] Ayres JS, Schneider DS. Tolerance of Infections. In: Paul WE, editor. Annu Rev Immunol Vol 30. Palo Alto: Annual Reviews; 2012. p. 271–94. 10.1146/annurev-immunol-020711-075030 22224770

[44] Rowland RRR, Lunney J, Dekkers J (2012). Control of porcine reproductive and respiratory syndrome (PRRS) through genetic improvements in disease resistance and tolerance. Front Genet.

[45] Zimmerman JJ, Yoon KJ, Pirtle EC, Wills RW, Sanderson TJ, McGinley MJ (1997). Studies of porcine reproductive and respiratory syndrome (PRRS) virus infection in avian species. Vet Microbiol.

[46] Albina E (1997). Epidemiology of porcine reproductive and respiratory syndrome (PRRS): an overview. Vet Microbiol.

[47] de Pax X, Vega D, Duran C., Angulo J. PRRS prevalence in Europe: Perception of the pig veterinary practitioners—PRRS.com. ESPHM 2015. 2015. p. 1; Accessed on 22 Sep 2015.

[48] Bishop SC, Woolliams JA (2014). Genomics and disease resistance studies in livestock. Livest Sci.

[49] Bishop SC (2012). A consideration of resistance and tolerance for ruminant nematode infections. Front Genet.

[50] Mauricio R, Rausher MD, Burdick DS (1997). Variation in the defense strategies of plants: are resistance and tolerance mutually exclusive?. Ecology.

[51] Lough G, Kyriazakis I, Bergmann S, Lengeling A, Doeschl-Wilson AB (2015). Health trajectories reveal the dynamic contributions of host genetic resistance and tolerance to infection outcome. Proc Biol Sci.

[52] Bisset SA, Morris CA (1996). Feasibility and implications of breeding sheep for resilience to nematode challenge. Int J Parasitol.

[53] Herrero-Medrano JM, Mathur PK, Napel J ten, Rashidi H, Alexandri P, Knol EF, et al. Estimation of genetic parameters and breeding values across challenged environments to select for robust pigs. J Anim Sci. 2015;93:1494–502. 10.2527/jas.2014-8583 26020171

[54] Rashidi H (2016). Breeding against infectious diseases in animals.

[55] Allen JE, Sutherland TE (2014). Host protective roles of type 2 immunity: parasite killing and tissue repair, flip sides of the same coin. Semin Immunol.

[56] Doeschl-Wilson AB, Bishop SC, Kyriazakis I, Villanueva B (2012). Novel methods for quantifying individual host response to infectious pathogens for genetic analyses. Front Genet.

[57] Gopinath S, Lichtman JS, Bouley DM, Elias JE, Monack DM (2014). Role of disease-associated tolerance in infectious superspreaders. Proc Natl Acad Sci USA.

[58] Anacleto O, Garcia-Cortés LA, Lipschutz-Powell D, Woolliams JA, Doeschl-Wilson AB (2015). A novel statistical model to estimate host genetic effects affecting disease transmission. Genetics.

