# Supplementary material for: Use of multi-trait and random regression models to identify genetic variation in tolerance to porcine reproductive and respiratory syndrome virus
Source: Genet Sel Evol. 2017 Apr 19;49:37. doi: 10.1186/s12711-017-0312-7 (PMC5396128; doi:10.1186/s12711-017-0312-7)
Supplement: Supplementary file 1 — Additional file 1. Simulating ADG in absence of infection. The additional file provides a detailed methodology on how performance in absence of infection was simulated. [file 12711_2017_312_MOESM1_ESM.docx]

**Simulating ADG in absence of infection**

To assess the impact of additional measures of performance in the absence of infection (i.e. true intercept) on genetic parameter estimates for tolerance, average daily gain in absence of infection (ADG_0_) was simulated for paternal half-sibs of the infected individuals in the dataset. ADG^0^_21_ and ADG^0^_42_ were simulated for both observation periods from 0-21dpi and from 0-42dpi, respectively, assuming a heritability of 0.4 [1] and a genetic variance half of that of the genetic variance for average daily gain under infection ADG_I_ (where ADG_I_ stands for ADG_21_ and ADG_42_, respectively). A smaller variance was assumed for ADG_0_ since part of the variation in ADG_I_ is due to different levels of VL. Since it was expected that genetic variance estimates for tolerance depend on the genetic relationship between ADG_0_ and ADG_I_ (see Figure 1 in main manuscript), different values of genetic correlations (r_g_) between ADG_0_ and ADG_I_ were simulated representing weak, moderately low, moderately strong and strong genetic relationships between the traits with corresponding values of 0.05, 0.30, 0.60 and 0.90, respectively.

The simulated values for ADG^0^_21_ and ADG^0^_42_ were produced as follows:

**Step 1:** Calculate sire breeding values for growth under infection (ADG_I_)

Firstly, sire breeding values were calculated for ADG under infection (ADG_I_) from 0-21 or 0-42 dpi (ADG_21_ and ADG_42_, respectively) using sire estimated breeding values (EBV_S_) from the level model, described in main text, using the following equation:

${BV}_{s}^{{ADG}_{I}}={EBV}_{S}^{{ADG}_{I}}+PE$, [1]

Where the prediction error ($PE$) was sampled using:

$PEV =\sigma_{A_{{ADG}_{I}}}^{2}-var \left( EBV\left( {ADG}_{I} \right) \right)$,

where $\sigma_{A_{{ADG}_{I}}}^{2}$ is the additive genetic variance of ADG under infection, and sire EBVs were acquired from the level model.

**Step 2:** Calculate sire breeding values for growth in the absence of infection (ADG_0_)

Breeding values were calculated for ADG in the absence of infection (ADG_0_) for 0-21 or 0-42 dpi (ADG^0^_21_ or ADG^0^_42_, respectively) using the following equation:

${BV}_{s}^{{ADG}_{0}}=b_{{BV}^{{ADG}_{0}},{BV}^{{ADG}_{I}}}{BV}_{s}^{{ADG}_{I}}+{RES}_{1}$, [2]

Where the regression coefficient $b_{{BV}^{{ADG}_{0}},{BV}^{{ADG}_{I}}}$ was calculated as:

$$r_{g}\frac{\sigma_{A_{{ADG}_{0}}}}{\sigma_{A_{{ADG}_{I}}}}$$

where $r_{g}$ is the simulated genetic correlation between ADG_0_ and ADG_I_ (simulated as either 0.05, 0.30, 0.60 or 0.90), and $\sigma_{A_{{ADG}_{0}}}$ and $\sigma_{A_{{ADG}_{I}}}$ are the square roots of the additive genetic variance in ADG_0_ and ADG_I_, respectively, and the residual (${RES}_{1}$) was sampled from:

$$N(0,\left( 1-r_{g} \right)^{2}\sigma_{A_{{ADG}_{0}}}^{2})$$

**Step 3:** Calculate phenotypic values for growth without infection for progeny (half sibs) (P_Prog_)

Finally, phenotypic values of growth without infection were simulated for one paternal half-sib of each infected individual in the data set , as follows:

${ADG}_{0}=\frac{1}{2}{BV}_{s}^{{ADG}_{0}}+{RES}_{2}$, [3]

where ${RES}_{2}$ was sampled from:

$N(0,(1-\frac{1}{4}h^{2})\sigma_{p_{{ADG}_{0}}}^{2}$)

where phenotypic variance of ADG­_0_ ($\sigma_{p_{{ADG}_{0}}}^{2}$) was calculated as:

$$\frac{\sigma_{A_{{ADG}_{0}}}^{2}}{h_{{ADG}_{0}}^{2}}$$

Following this approach, phenotypes for ADG^0^_21_ and ADG^0^_42_ were generated for one half-sib per infected individual in the dataset, thus doubling the size of the dataset. For each simulated population, 10,000 replicates were generated. Table 1 below shows the means (and standard errors) for the simulated true ADG_0_ breeding values, the genetic variances and correlations between true breeding values of ADG_0_ and ADG_I_.

**Table 1.** Mean sire breeding value and genetic variance of simulated performance in absence of infection (ADG_0_), where r_g_ is simulated genetic correlation between ADG_0_ and ADG under infection (ADG_I_) for the 21- or 42-day observation periods. Correlations between breeding values of ADG_0_ and ADG_I_ are also shown. Standard error over 10,000 replicates is shown in brackets.

| **r_g_** | **Mean (**$\mathbf{BV}_{\mathbf{s}}^{\mathbf{ADG}_{\mathbf{0}}}$**)** | $\boldsymbol{\sigma}_{\mathbf{A}_{\mathbf{ADG}_{\mathbf{0}}}}^{\mathbf{2}}$ | **Correlations**  **between** $\mathbf{BV}_{\mathbf{s}}^{\mathbf{ADG}_{\mathbf{0}}}$ **and** $\mathbf{BV}_{\mathbf{s}}^{\mathbf{ADG}_{\mathbf{I}}}$ |
| --- | --- | --- | --- |
| **21 day observation period** | | | |
| **0.05** | 2.34E-04 (1.20E-04) | 9.36E-04 (1.55E-05) | 0.05 (0.14) |
| **0.30** | 1.04E-03 (1.15E-04) | 1.26E-03 (1.62E-05) | 0.30 (0.12) |
| **0.60** | 2.07E-03 (9.41E-05) | 3.39E-03 (1.61E-05) | 0.60 (0.08) |
| **0.90** | 3.01E-03 (5.23E-05) | 3.44E-03 (1.75E-05) | 0.90 (0.02) |
| **42 day observation period** | | | |
| **0.05** | 1.81E-04 (1.47E-04) | 1.32E-03 (2.33E-05) | 0.05 (0.14) |
| **0.30** | 1.46E-03 (1.40E-04) | 1.35E-03 (2.32E-05) | 0.30 (0.12) |
| **0.60** | 2.83E-03 (1.16E-04) | 1.51E-03 (2.65E-05) | 0.60 (0.08) |
| **0.90** | 4.34E-03 (6.42E-05) | 1.44E-03 (2.76E-05) | 0.90 (0.02) |

**REFERENCES**

1. Chen P. Genetic improvement of lean growth rate and reproductive traits in pigs. [cited 2016 Nov 14]; Available from: http://lib.dr.iastate.edu/rtd
